# Who Owns the Medical Paper?

**Published:** 1897-01

**Authors:** Geo. M. Gould

**Affiliations:** 119 South 17th St.; Philadelphia


### CORRESPONDENCE.

### WHO OWNS THE MEDICAL PAPER?

PHILADELPHIA, December 15, 1896.

*To the Members of the Medical Profession:*

I would be pleased to have an expression of opinion from you, either personally or through some medical journal, as to the relations of the lay-publishing firms of medical journals and the profession. The request is suggested by the fact that Messrs. Wm. Wood & Co., of New York, refuse to permit the editors of *The American Year-Book of Medicine and Surgery* to use in our abstracts of Medical Progress articles and illustrations first printed in the *Medical Record* and the *American Journal of Obstetrics.*

This decision seems to be wrong for the following reasons:

1. It prevents the dissemination of medical knowledge. The *Year-Book* condenses, systematizes, and criticises the year's medical work in a shorter space and more permanent manner than the journals, and has thousands of readers no single journal can claim or hope to reach. Every physician writes and publishes articles in order that every member of the profession may, if possible, learn of his work, and that science and progress may thus be furthered and humanity benefited. To interfere with such dissemination of our literature in reputable publications is, I think, discourteous and unjust to the profession and an injury to medical science.

2. This injustice and injury to medicine become all the more striking when physicians do not receive a cent of pay for contributions, from the publication of which the lay-publisher is supposed to make considerable financial profit.

3. No other publishers in the world, not even those who pay authors for their contributions, have in the least objected to our reproduction of quotations, abstracts, and illustrations from their journals.

Do you wish to limit the dissemination of your contributions to medical science by such an exclusion of them on the part of publishers from reputable publications? *Is this literature the property of yourself and of the profession, or not?* Does your gift of it to a journal make it forever the private property of the publishers of that journal? Is it not rather a loan for temporary use only?

Will you not hereafter demand that there be printed with your article a statement that the right of abstracting the text or reproducing illustrations is guaranteed?

Sincerely yours,

*119 South 17th St.* GEO. M. GOULD, M.D.

---

### THE MURPHY BUTTON.

The *New York Polyclinic*, speaking of the use of the Murphy button in Germany, says that Schede and his assistants were able to obtain results in twenty-five cases which they could not have hoped for had the suture been employed. Five of the operations were gastro-enterostomies for benign causes, and all of the patients recovered. The same operation upon nine patients with carcinoma of the pylorus was followed by death in six cases; of six cases of carcinoma of the large intestine, three recovered; of four cases of strangulated gangrenous hernia, two recovered. In all these cases intestinal resection was performed. In a patient who sustained several wounds of the small intestines from a stab, 64 ctm. of ileum were resected, and the ends united by Murphy's button, recovery following. The length of time a Murphy button is retained has given rise to considerable discussion, and it is interesting to note that, although fourteen patients recovered, only seven of them passed buttons, as far as could be ascertained. The period varied from 12 to 34 days.—*American Jour. Surgery and Gynecology.*

---

### LOCAL TREATMENT FOR NEURALGIA.

The following is the latest Italian method for relieving neuralgia:

℞ Menthol,

Guaicol, aa .................................................. ʒ i.

Alcohol.............................................................. ʒ xviii.

M.

S.—A drachm of this mixture is to be rubbed lightly into the parts where pain is present two or three times a day. In the intervals a cotton dressing is employed.

—*Codex Medicus.*

